# Burns in Early Childhood: Age-Specific Causes, Risks, Management, and Implications—A Narrative Review

**DOI:** 10.3390/children12111424

**Published:** 2025-10-22

**Authors:** Gloria Pelizzo, Valeria Calcaterra, Carlotta Paola Maria Canonica, Vittoria Carlotta Magenes, Michela Marinaro, Eleonora Durante, Erika Cordaro, Gianvincenzo Zuccotti

**Affiliations:** 1 Department of Biomedical and Clinical Science, University of Milan, 20157 Milan, Italy; gloria.pelizzo@unimi.it (G.P.); gianvincenzo.zuccotti@unimi.it (G.Z.); 2 Pediatric Surgery Department, Buzzi Children’s Hospital, 20142 Milan, Italy; carlotta.canonica@unimi.it (C.P.M.C.); michela.marinaro@unimi.it (M.M.); eleonora.durante@unimi.it (E.D.); 3 Pediatrics and Adolescentology Unit, Department of Internal Medicine, University of Pavia, 27100 Pavia, Italy; erika.cordaro01@universitadipavia.it; 4 Pediatric Department, Buzzi Children’s Hospital, 20142 Milan, Italy; vittoria.magenes@unimi.it

**Keywords:** burns, early childhood, prevention, outcome, pediatrics, children

## Abstract

Burn injuries represent a significant global burden, with children under the age of five among the most vulnerable groups. This narrative review will explore the main causes of burns in early childhood (under 6 years of age), the associated risks, current treatment approaches, and the long-term implications of these injuries. It will also highlight areas where further research is needed to improve prevention and management strategies for burns in this vulnerable population. Results showed that burns in children under six years old represent a significant clinical and preventive challenge, with physical, psychological, and social implications. Research has identified common causes, particularly scalds from hot liquids, while advancing innovative treatments such as bioengineered skin substitutes, virtual reality, and telemedicine. Preventive interventions at the household and community levels have also proven effective. However, major limitations remain: studies often lack age-specific focus, rely on retrospective data, underrepresent low-resource settings, and lack standardized protocols. To improve outcomes, future research must adopt a more targeted, multidisciplinary approach and address long-term physical and psychological effects to ensure comprehensive, age-appropriate care.

## 1. Introduction

Burn injuries represent a significant global burden, with children under the age of five among the most vulnerable groups [1,2,3]. According to the World Health Organization, burns are the fifth most common cause of non-fatal childhood injuries, with children under five years old having the highest burn incidence. In Europe, they result in about 6000 pediatric hospitalizations every year [1]. In the United States, children under six represent 57% of pediatric burn cases treated in emergency departments [4]. In the WHO Western Pacific Region, which includes parts of Asia, the incidence of burns requiring medical care is nearly twenty times higher than in the Americas [1]. In the WHO African Region, children under five have more than twice the global average incidence of burn-related deaths [1,5]. In high-income countries, scalds from hot liquids are the most frequent cause, accounting for over 60% of burn injuries in children under five [2,4]. In contrast, in low- and middle-income countries, especially in Sub-Saharan Africa and South Asia, open flames, hot objects, and unsafe cooking practices and limited access to prevention tools contribute significantly to the risk of severe harm among children [5].

Burns in early childhood are among the leading causes of injury and physical harm. This age group is particularly at risk due to their thinner skin and natural curiosity, which drives them to explore dangerous situations, such as touching hot surfaces, boiling liquids, and sharp objects. Furthermore, young children cannot prevent accidents on their own and rely entirely on adults for their safety [6,7].

Burn patients have a wide spectrum of injury severity and diverse outcomes, ranging from superficial burns to deep, large body surface area burns which are profoundly life-changing, affecting physical and psychological systems [8].

In children, systemic effects of burns typically develop when the total body surface area (TBSA) burned exceeds approximately 15–20% [8,9,10]. Thermal injury triggers capillary leakage and massive plasma loss into the interstitial space, resulting in hypovolemia, edema, and burn shock. Due to their higher surface-area-to-mass ratio and limited circulatory reserve, children are particularly vulnerable to fluid loss [9,11], which can begin at TBSA values as low as 10–15%. In parallel, tissue damage and systemic inflammation promote the generation of reactive oxygen species (ROS) and disrupt antioxidant defenses, contributing to endothelial dysfunction, delayed wound healing, and organ complications [2,12,13,14,15]. Systemic inflammatory and metabolic responses become significant beyond 20% TBSA; however, in younger children, the clinical threshold for a systemic response may be even lower (≥15%), requiring early intensive care. Managing and monitoring burns in young children requires careful, specialized care that takes into account their unique physiological characteristics to minimize complications [8,9].

Current evidence indicates that over 70% of burn injuries in children under 6 years of age are preventable through appropriate home safety measures, continuous adult supervision, and targeted educational programs for parents and caregivers [6,7,10]. This striking statistic underscores the urgency and importance of further studying this topic, not only to improve clinical outcomes, but to implement effective prevention strategies that can save lives and reduce suffering.

This review will examine the main causes of burns in early childhood (under 6 years of age), associated risk factors, current treatments, and long-term implications. Understanding the unique aspects of wound healing in children is essential to emphasize the need for age-specific approaches that improve outcomes and prevent complications; for this reason, physiological differences will be specifically addressed. The review will also identify areas where further research is needed to enhance prevention and management strategies. A comprehensive understanding of these elements is crucial to guide public health interventions, inform caregiver education, improve clinical training, and support psychosocial recovery and rehabilitation in pediatric burn patients.

## 2. Methods

A narrative review was conducted to explore the age-specific causes, physiological implications, and management strategies of burn injuries in pediatric age, focusing on the challenges in children under 6 years of age. The cut-off at 6 years was chosen to distinguish early childhood as a distinct developmental stage characterized by specific behavioral, cognitive, and physiological vulnerabilities to burns. At this age, children exhibit limited motor coordination, immature risk perception, and high dependency on caregivers, which increase their exposure to domestic hazards. In addition, their skin is thinner and more sensitive, resulting in deeper injuries from the same thermal exposure compared to older children [8,16].

A comprehensive literature search was carried out using PubMed and Scopus databases. Keywords and search terms included: burns, burn injuries, early childhood, children, pediatric, severe burns, scalds, risk factors, metabolic response, hormonal changes, oxidative stress, stress response, nutritional support, and management. Boolean operators (AND/OR) were applied to structure the query. No restrictions were applied regarding geographic origin.

Four independent reviewers (C.P.M.C., V.C.M., M.M., and E.D.) conducted a two-step screening process: title and abstract review followed by full-text screening. Discrepancies during screening were resolved through discussion or consultation with a senior reviewer (V.C).

Inclusion criteria were as follows: studies involving pediatric patients with burn injuries in early childhood, with particular attention to the influence of age on the causes, physiological responses, and management of burns; publications in English between 2005 and 2025; and article types including original research, clinical trials, meta-analyses, and review articles. Eligible studies addressed burn causes, risk factors, systemic or physiological responses, and therapeutic or nutritional interventions specific to this early age group. Exclusion criteria included case reports, case series, editorials, letters, or conference abstracts, as well as articles lacking clinical, physiological, or management relevance to pediatric burns. Although the review primarily focused on children in early childhood, studies involving older pediatric, adolescent, or adult populations were also included when they provided age-comparative data or mechanistic insights relevant to understanding burn pathophysiology in young children.

## 3. Search Results

The initial search yielded a total of 482 records. After screening titles and abstracts, 301 articles were excluded due to duplication or irrelevance. A total of 181 full-text articles were assessed for eligibility. Among these, 119 articles met the predefined inclusion criteria for full-text analysis, and 100 articles were ultimately included in the manuscript. Reference lists of the included studies were further examined to identify additional pertinent publications.

## 4. Distinct Causes and Risk Factors

### 4.1. Specific Causes of Burns in Children Aged 0–6 Years

Burns represent a frequent form of trauma in early childhood (ages 0 to 6), characterized by injury mechanisms and anatomical distributions that differ from those in older age groups. The majority of these burns are thermal, with scalds from hot liquids constituting the predominant etiology [2].

These injuries primarily result from exposure to boiling water, hot beverages, cooking oil, and other domestic liquids, often due to accidental spillage or children pulling containers toward themselves [3]. The most commonly affected anatomical sites are the upper extremities (hands and forearms), involved in 68% of cases, followed by the face (15%) and lower extremities (10%). The remaining 7% involve the chest and other body regions [4].

Data from the U.S. National Electronic Injury Surveillance System (NEISS) reported an estimated 11,028 annual cases of scald burns in children under 3 years of age, highlighting the significant prevalence of this injury pattern in early childhood [10]. Children under five years of age in the WHO African Region have more than twice the incidence of burn deaths compared to the global average for this age group, and boys under five years living in low- and middle-income countries of the WHO Eastern Mediterranean Region are almost twice as likely to die from burns as boys in the WHO European Region [1,5].

Superficial second-degree burns represent the majority of scald injuries, while a smaller proportion progress to deep second-degree or third-degree burns, which often require surgical interventions such as debridement or skin grafting [1,2,3]. The incidence of burn injuries requiring medical care is nearly twenty times higher in the WHO Western Pacific Region than in the WHO Region of the Americas [5].

In high-income countries, although flame burns are less common in children under 6 years of age, they are often more severe and associated with higher mortality. Data involving burn centers across Europe reported that flame burns accounted for approximately 15–20% of severe pediatric burn cases [1,8]. These injuries usually result from residential fires or the unsupervised handling of flammable materials (such as matches and lighters) [8]. The average proportion of flame burns among children in low- and middle-income countries within the WHO African Region ranges from approximately 30% to 40%, depending on the context. Recurrent risk factors include household overcrowding, unemployment and limited access to safe cooking facilities [5].

Chemical and electrical burns, though less frequent, comprise a significant proportion (5–10%) of pediatric burn injuries [17]. Common causes include accidental contact with household cleaning agents or unprotected electrical sources. In children under 6 years of age, electrical burns are predominantly caused by low-voltage indoor sources such as unsecured wall sockets, damaged electrical cords, or contact through the mouth or hands [18,19]. These injuries usually involve a small total body surface area but often affect functionally and aesthetically critical regions, including the lips, fingers, and oral commissures. Ref. [18] Although generally localized, they may cause deep tissue damage at the contact site, leading to necrosis, scarring, and contractures. The long-term consequences are primarily functional and aesthetic, frequently requiring reconstructive treatment and multidisciplinary follow-up [18,19]. The extent of injury depends on factors such as contact duration, current path, and tissue resistance, with oxidative stress and membrane disruption contributing to delayed healing [12,13,14,20,21].

### 4.2. Environmental and Behavioral Risk Factors

The home environment, particularly the kitchen and dining areas, constitutes the principal setting for burn injuries in children. According to data from HealthLinkBC, over 50% of pediatric burn injuries occur in the kitchen, predominantly due to accidental spillage of hot liquids, contact with heated surfaces, or exposure to open flames [6]. These findings underscore the critical need for targeted home safety interventions in areas frequently accessed by young children.

Socioeconomic status is a well-established determinant of burn risk, particularly in low- and middle-income countries [5]. Studies conducted in low-income urban settings have shown that children from families of lower socioeconomic strata are three times more likely to sustain burn injuries compared to their counterparts from middle- or high-income families [22]. This disparity is frequently attributed to substandard housing, limited access to safety devices, and reduced exposure to injury prevention education [1,5].

Lapses in adult supervision constitute another major behavioral risk factor. Epidemiological studies report a 30–40% increase in pediatric burn incidence associated with inadequate supervision or brief moments of caregiver distraction. Young children, due to their limited risk awareness, are particularly vulnerable during routine household activities when left unsupervised [7].

### 4.3. Individual and Physiological Risk Factors

Age plays a critical role in burn risk. According to the CDC, children aged 1 to 3 years exhibit the highest incidence rates, with an estimated 15–20 cases per 1000 children annually [23]. This age group corresponds to a developmental phase marked by increasing motor autonomy but insufficient hazard perception.

Additional risk factors include motor and sensory impairments, which significantly elevate burn susceptibility. A 10-year retrospective analysis revealed that children with disabilities had a 2- to 3-fold higher risk of burn injuries compared to their non-disabled peers, primarily due to impaired hazard avoidance and increased dependency on caregivers for daily activities [8].

In a cohort of 1498 pediatric burn patients treated between 2015 and 2020, 32.5% had burns involving more than 20% of the total body surface area (TBSA), a threshold associated with increased clinical severity and complication rates. The overall mortality rate was estimated at 2.5%, with higher rates observed in patients with extensive TBSA involvement [24].

### 4.4. Cultural and Social Considerations

The incidence and etiology of pediatric burns are strongly influenced by cultural practices and geographic context. In many regions of Asia and Africa, the widespread use of open-flame cooking, wood stoves, and solid fuels significantly increases the risk of flame burns and contact with hot surfaces [8]. In Middle Eastern countries, particularly Syria, petroleum-based fuels are commonly used for domestic heating and are often stored in areas easily accessible to children. As a result, accidental contact and ignition frequently lead to severe burn injuries. In addition, in many low-income settings, electric heaters are used in children’s bedrooms for heating purposes; these devices may accidentally tip over and cause house fires. Conversely, in higher-income or urban settings, pediatric burns are more often associated with scalds from hot liquids, kitchen appliances, or recreational activities, as families in these environments generally benefit from safer cooking facilities, improved housing infrastructure, and greater awareness of burn prevention and safety measures [9,10].

Socioeconomic disparities further affect the efficacy of burn prevention campaigns and the implementation of safety regulations. In low- and middle-income countries (LMICs), the incidence of pediatric burns is up to ten times greater than in high-income settings, contributing to a higher burden of morbidity and mortality. In several regions of Southeast Asia and sub-Saharan Africa, burns are among the leading causes of death and long-term disability in children under 6 years of age [1,5,25].

Current evidence indicates that over 70% of burn injuries in children under 6 years of age are preventable through appropriate home safety measures, continuous adult supervision, and targeted educational programs for parents and caregivers [1,10]. Effective preventive strategies include the use of stove guards and pot handle covers, regulation of domestic hot water temperatures, and the design of child-safe environments that minimize exposure to thermal and chemical hazards.

## 5. Physiological Differences Between Children and Adults in Burn Management

Pediatric burn patients differ substantially from adults in several physiological and anatomical parameters that directly influence treatment and recovery, Table 1. Understanding the unique aspects of wound healing in children remains a key priority for improving outcomes and preventing complications such as fluid imbalance, infection, and scarring [26,27,28].

In pediatrics, size, site, and depth of the burn are crucial factors affecting prognosis [28,29], but age-related differences further modify the clinical course. Children have a larger body surface area relative to body mass, thinner skin, smaller airways, and lower circulating blood volume, which make them more susceptible to hypovolemia, airway compromise, and hemodynamic instability after a burn injury [30,31].

Moreover, pediatric patients experience greater challenges in maintaining thermal balance and are more susceptible to rapid fluid loss due to their higher surface-area-to-volume ratio and immature thermoregulatory mechanisms [29,32]. The thinner epidermis and dermis in young children also contribute to deeper tissue damage from the same thermal exposure compared to adults, prolonging healing time and increasing the risk of hypertrophic scarring. Finally, the relative immaturity of the immune system, characterized by less efficient innate and adaptive responses, makes children more prone to infection and systemic inflammatory complications [33,34].

## 6. Hypermetabolic Response and Post-Burn Nutritional Needs in Children

The body’s response to a major burn can persist up to a year and delayed growth up to years postburn [35,36]. Indeed, in case of burns at first a phase of initial metabolism depression with tissue hypoperfusion occurs, a hypermetabolic and hyperdynamic circulatory phase ensues [37]. Without proper care, this leads to a chain of adverse events as weight loss, muscle and bone catabolism, retarded growth and immunosuppression [35,36]. Thus, modulation of the burn patients’ nutrition and metabolic response are key factors in their recovery and rehabilitation and prevention of long-term sequelae in growth and neurodevelopment [37].

### 6.1. Hypermetabolic Response in Pediatric Burn Patients

Pediatric burn patients exhibit a distinct and exaggerated hypermetabolic response compared to adults, primarily due to their limited metabolic reserves and ongoing developmental needs [37,38]. Children, particularly under the age of 5, are at increased risk of this condition due to greater evaporative and fluid losses (related to the higher surface area-to-body mass ratio), immature thermoregulation and endocrine systems, higher baseline metabolic rate compared to adults (estimated at 50–70 kcal/kg/day even in healthy states) and limited glycogen and fat stores [39]. These factors not only amplify the hypermetabolic response but also increase the risk of hypoglycemia, lean body mass depletion, and immune dysfunction [39].

This hypermetabolic response, that is an adaptive but deleterious physiological reaction, begins within hours of burn injury [40] and leads to significant challenges in clinical management, especially regarding nutrition, which is a cornerstone of recovery [37]. In children, this response is characterized by an increase in resting energy expenditure (REE) [41], profound protein catabolism and muscle wasting [41], elevated cardiac output and oxygen consumption [42], persistent insulin resistance and glucose intolerance [42], increased core body temperature and delayed return to metabolic homeostasis [42].

Interestingly, in a prospective study of 220 pediatric patients with burns >40% of total BSA, Jeschke et al. observed that REE remained elevated at up to 160% of predicted values for over 12 months post-injury [41]. The same cohort demonstrated significant alterations in mitochondrial function and muscle protein turnover, underscoring the severity of the metabolic disruption [41]. The hypermetabolic response is mediated by elevated levels of catecholamines, cortisol and glucagon, all of which stimulate lipolysis, proteolysis, and gluconeogenesis [42]. In addition, also cytokines such as interleukin-6 (IL-6) and tumor necrosis factor-alpha (TNF-α) contribute to systemic inflammation, further exacerbating catabolic effects [39,42].

### 6.2. Nutritional Needs and Support to Modulate Hypermetabolic Response

In burn care, nutrition is an important therapeutic intervention in the modulation of the hypermetabolic response [43,44]. The aims of a proper nutritional intervention are to reverse the catabolism, support the immune system and the wound healing process and enable normal growth and neurocognitive development [45].

Estimating energy requirements is challenging, since standard predictive formulas are often inaccurate in burn cases. Indirect calorimetry is considered the gold standard, but when unavailable, equations like the Schofield formula can be used [45], keeping in mind that caloric needs are generally very high, around 1500 to 2000 kcal/m^2^ of body surface area or 1.5 to 2 times the resting metabolic rate, depending on age and burn severity [45]. Protein intake should represent 20–25% of total calories, amounting to about 2.5–4 g/kg/day in children, as protein synthesis is fundamental for tissue repair and immune competence [37,46]. Carbohydrates are the preferred energy source, covering 60–70% of total caloric needs but not exceeding 400 g/day to avoid metabolic complications such as hyperglycemia [37]. Fats should account for 20–25% of calories, with a preference for low-fat and omega-3–rich formulations to minimize hepatic steatosis, immune suppression, and delayed healing [37,43]. Micronutrient requirements, particularly vitamins A, C, and D, as well as zinc, selenium, copper, and iron, increase substantially, as they are essential for collagen synthesis, epithelial regeneration, and immune defense [37,43,46].

Although oral feeding is ideal, it is often difficult in acute burn patients due to clinical complications; therefore, early enteral nutrition, started within 24–48 h (and preferably within 4 h of admission), is strongly recommended for its safety and benefits in reducing infections, preserving gut integrity, and shortening ICU stay [37,47]. Total parenteral nutrition should be reserved for patients who cannot tolerate enteral feeding, as it carries higher risks such as hepatic dysfunction, infection, and immune suppression [37,47].

## 7. Pediatric Burn Management

Pediatric burns require a comprehensive multidisciplinary approach that integrates medical, surgical, and innovative therapies. A specialized team should include pediatric burn surgeons, anesthesiologists, pain management specialists, physiotherapists, psychologists, social workers, occupational therapists, dietitians, and nurses. These professionals play a vital role in addressing the complex and evolving needs of young burn patients and in improving their long-term recovery and quality of life. The primary goals of a multidisciplinary approach are to optimize wound healing, prevent infections, manage pain effectively, and enhance both functional and psychological outcomes.

### 7.1. Early Intervention in Burn Management

Burns in young children must be approached in an urgent and precise way, in order to have better outcomes and minimize complications. Early management is mandatory, starting from resuscitation protocols and taking in charge pediatrics patients with specialized protocols [30,48].

Total Body Surface Area (TBSA) >10–15% requires promptly fluid resuscitation, following pediatric protocols tailored on this particular population [49,50]. Fluid replacement should be balanced in order to avoid over or under filling, to prevent complications such as shock or fluid overload and should be guided by cardiovascular monitoring [11].

Initial management must include early wound care and prevention of hypothermia, to prevent infectious risks and shock appearance [50,51].

Antimicrobial dressings remain the standard of care for reducing bacterial colonization and infection risk, critical factors given children’s heightened susceptibility to sepsis and delayed healing [52,53].

Burn care should include protocols to avoid infections. Surgical or enzymatic wound excision and grafting are demonstrated to reduce the duration of open wounds, thereby decreasing the risk of invasive infections and systemic inflammatory response [54,55].

Innovative therapeutic modalities are increasingly being explored in pediatric burn care. Regenerative medicine, particularly stem cell–based therapies, has shown promising potential in enhancing wound healing and tissue repair in burn injuries. These approaches have been reported to accelerate re-epithelialization by up to 25% compared to conventional treatments and to improve the quality of scar formation. In experimental models of severe scald burns, subdermal injection of adipose-derived stem cells has demonstrated improvements in wound healing by up to 30%. However, most of the available evidence remains preliminary, especially in pediatric populations.

Autologous skin grafting remains the gold standard for deep burns, but donor site limitations have prompted the development of advanced alternatives such as cultured epithelial autografts, dermal substitutes, and bioengineered skin equivalents. Composite bilayer materials, combining dermal and epidermal analogs, have demonstrated improved mechanical stability, reduced contracture formation, and superior long-term cosmetic results [56,57]. Similarly, bioengineered skin substitutes composed of biodegradable scaffolds seeded with dermal and epidermal cells facilitate cellular proliferation, promote vascularization, and support re-epithelialization, thereby reducing infection rates and long-term scarring, an especially important outcome in growing children [58,59,60].

In pediatric burn patients, the Meek micrografting technique has emerged as a highly effective autografting option when donor skin availability is limited [57,61,62,63,64]. This technique allows for substantial expansion ratios while maintaining excellent graft take, rapid epithelialization, and satisfactory aesthetic outcomes [57]. Several studies have confirmed its efficacy and safety in extensive pediatric burns, highlighting improved healing times and reduced need for regrafting compared with traditional mesh grafting [57,63,64]. In particular, the modified Meek technique has proven especially beneficial in very young children, providing reliable wound coverage and facilitating survival in major burns exceeding 60–70% TBSA [57,61,65].

Additionally, negative pressure wound therapy (NPWT) has been associated with a 15–20% increase in skin graft take rates and a reduction in postoperative edema, representing a valuable adjunctive tool in the management of complex wounds [66,67,68].

Future perspectives include the integration of 3D bioprinting technologies for precise cellular deposition and replication of native skin architecture, as well as the use of growth factor–enriched scaffolds and exosome-based treatments aimed at modulating inflammation and promoting tissue regeneration [69,70,71,72].

### 7.2. Pain Management and Psychological and Rehabilitative Support

Initial management should also include an effective pain management, considering the strong impact of burns and to prevent potential long-term psychological issues [73].

Prevention of pain is crucial to allow a correct healing and also to head off long-term psychological sequelae correlated to stress and hospitalization stay. Pain management strategies have evolved to incorporate multimodal analgesia, including opioid and non-opioid agents tailored to pediatric pharmacodynamics, alongside adjuncts such as regional anesthesia techniques. Recent studies have demonstrated the value of non-pharmacological interventions, including distraction techniques (e.g., virtual reality, video games, interactive applications), which significantly reduce pain perception and anxiety in young patients [74,75,76].

Starting from a young age, psychological support is necessary to avoid emotional distress, anxiety and risk of post-traumatic stress disorder (PTSD), which can adversely affect recovery and quality of life. Within the first year post-injury, 30–45% of children develop post-traumatic stress disorder (PTSD), and 25–35% exhibit symptoms of anxiety or depression [77,78]. Younger age at the time of injury and adverse family dynamics are recognized risk factors for poorer psychological outcomes. Evidence suggests that early psychological intervention can reduce the incidence of long-term mental health disorders by up to 40%, thereby facilitating more favorable psychosocial adjustment [79].

Early involvement of mental health professionals facilitates the identification and management of these issues through family-centered counseling, cognitive-behavioral therapy, and age-appropriate coping strategies [79,80].

Children’s path into burn care should include from the initial management rehabilitation interventions, aiming to restore functionality and to reduce psychological impact of the traumatic event.

Rehabilitation offers a range of benefits, including improvements in mobility and physical function, reduction of pain, and enhancement of overall quality of life [81,82,83,84]. It supports the recovery of independence in daily activities, promotes mental health and psychological well-being, and helps prevent or treat scarring and contractures. In children, rehabilitation aims not only to restore physical function but also to support growth and prevent deformities that could interfere with development. Early rehabilitation is particularly emphasized to minimize long-term complications.

Interventions often involve active participation of the family to ensure continuity of care at home and to foster psychological adaptation. Personalized programs are designed to address the child’s educational and social needs during recovery, ensuring that physical healing is accompanied by holistic support for overall development [81,82,83,84].

Tailored exercise programs, combined with splinting and positioning techniques, promote optimal scar remodeling and maintain mobility during the critical phases of wound healing [82].

## 8. Prevention Strategies

The domestic environment is the most common setting for burn injuries in young children, especially in the absence of proper safety measures, with kitchens and bathrooms being considered particularly hazardous areas. Hot liquid scalds are the most frequent type of burn, followed by contact burns from hot surfaces or objects [85]. Young children are highly vulnerable due to their nature. They approach the world with curiosity, have a poorly developed sense of danger and, therefore, a lower tendency to act appropriately to avoid or mitigate the risk of injury. Furthermore, it has been highlighted how parents often tend to underestimate the risks to which their children are exposed and the clinical consequences of a possible injury [86,87]. However, the prevailing perspective among burn care professionals is that most burn injuries, particularly those involving children, can be prevented [2] thus underlining how could prevention play a crucial role.

Although the effectiveness of prevention strategies has not been widely evaluated and the studies conducted show mixed results, there is considerable evidence supporting that prevention requires a systematic approach that integrates structural safety modifications, aimed at making the environment in which the child spends most of the day safer (passive prevention) and targeted educational efforts aimed at parents and caregivers, to encourage parental supervision and protection of children and in general to promote awareness in risk assessment and management (active prevention). Kendrick et al. reported that combined approaches including environmental and behavioral components are the most effective [88]. Phelan et al. [89] also confirmed the effectiveness and cost-efficiency of combining education and home safety interventions through randomized controlled trials, although analysis conducted by Turner et al. [90] reported insufficient evidence to determine whether interventions focused on modifying environmental home hazards reduce injuries and suggest further evaluations are needed.

Institutional guidelines for burn prevention [91,92] include critical changes in the environment to reduce burn risk in young children, such as: using rear stove burners; turning pot handles inward; shielding hot surfaces, fireplaces, stoves and ovens when in function; installing smoke detectors; adjusting water heaters to below 48 °C and regulating the temperature of water flowing from household taps; do not store flammable liquids in the kitchen. The Canadian government recommends, for example, the installation of automatic or anti-scald mixing valves to prevent scald injuries [93] when bathing the baby. Cagle et al. [94] reported that interventions such as the installation of safety devices, water temperature regulators, and stove guards are useful in reducing accidental injuries, although further investigations evaluating the long-term impact of preventive strategies are needed.

Educational programs tailored to children and their caregivers have also demonstrated strong impact in improving home safety behaviors. Kendrick et al. reported that approaches such as educational campaigns and safety product distribution reduce injuries significantly [95] as well as reported by Gielen et al. [96] and Cagle et al. [94]. The pilot study conducted by Corrarino et al. [97] and the “Childhood Burn Prevention Program” evaluated in Turkey [98] also showed that an educational program for families with young children significantly reduced risk factors and improved the adoption of preventive measures against household risks. Finally, the effectiveness of educational programs in promoting home safety was confirmed by Castaneda-Rodríguez et al. [99]. The study, evaluating the “No + Quema2” educational program between 2014 and 2022 to prevent burns in children under 9 years old, involved over 25,000 students and led to a significant reduction in pediatric burn incidence compared to the national average, with an estimated 3839 cases prevented.

Institutions work actively to enhance public knowledge and awareness about burns and its prevention, particularly in children. PRIUS project [100] (prevention of burn injuries during school age) was a national initiative promoted in 2011 in Italy funded by the Ministry of Health (CCM-National Centre for Disease Control and Prevention) and coordinated by the Italian National Health Institute (ISS). The initiative proposed the introduction of educational interventions in schools aimed at children, teachers and parents. The main activities included the distribution of interactive educational materials for children and the organization of training campaigns to spread and increase public awareness on the topic. Although it is no longer active, PRIUS project has contributed significantly to demonstrate the effectiveness of educational strategies in preventing the risk of burns in children and has highlighted the importance of collaboration between health and school institutions in promoting home safety strategies [100].

Generally, the guidelines aimed at educating parents report some preventive strategies such as: prepare the bathwater in advance before placing the baby in the tub and verify that the temperature of the bathwater does not exceed 37 °C to avoid the contact with excessively hot water; When holding the baby, do not carry hot food or liquids to prevent any risk of accidental spills or burns; avoid placing cups or plates with hot drinks or food near the edges of tables or shelves; store dangerous products in places that are not accessible to children and teach them to avoid hazardous materials; communicate to define of who will supervise the child. It is also important for caregivers to adjust their strategies as their child grows and develops to adapt them to new capabilities like reaching higher or climbing [101,102].

Although most evidence on burn prevention originates from high-income countries, the literature also highlights several strategies that may be applicable and adaptable to low- and middle-income settings, where the burden of pediatric burns is often higher. In these contexts, the implementation of preventive measures can be more challenging due to limited resources, reduced access to safety devices, lack of structured educational campaigns, and infrastructural barriers [1,2,3]. Nevertheless, community-based educational interventions, low-cost environmental modifications, and the active involvement of local stakeholders have been identified as promising and feasible approaches [5].

Educational efforts, delivered through schools, health workers, or community initiatives, combined with simple behavioral recommendations (e.g., keeping hot liquids out of reach and safer cooking practices) can help reduce the risk of burns, even in resource-limited settings. Training programs targeting caregivers, community health workers, and midwives have also been proposed as scalable strategies to raise awareness and promote safe practices within households [6,8,88,103,104,105].

It is essential to promote and evaluate context-specific prevention strategies tailored to the social, economic, and cultural conditions of low-resource environments. Further research is needed to assess the effectiveness and sustainability of these interventions and to support the development of integrated, evidence-based models of burn prevention globally.

## 9. Limitations

We chose to conduct a narrative review because this format allows for a broad and flexible synthesis of current knowledge, particularly in areas where the literature is heterogeneous in terms of study design, populations, and outcomes. However, when interpreting the results of this review, it is important to acknowledge several limitations. Unlike a systematic review, this narrative review offers a qualitative overview of selected studies, without adhering to formalized, standardized procedures. As a result, there is a risk of selection bias, and the scope of the review may be less exhaustive. Notably, the literature search was confined to PubMed and Scopus databases, which may have led to the omission of pertinent studies indexed elsewhere.

Additionally, although there is a growing interest in understanding burns in children under 6 years of age, current research still presents several limitations that hinder a comprehensive and age-specific approach, as the limited number of studies specifically addressing burn injuries in early childhood represents a significant gap in the literature. Many studies tend to group young children into broad pediatric categories, combining infants, toddlers, and preschoolers, which further limits the ability to identify age-related differences in burn mechanisms and outcomes. This practice may obscure important developmental and physiological differences that are crucial for understanding burn mechanisms and outcomes.

It is also important to note that most available data are derived from high-income countries, where reporting systems and access to specialized burn care are well established. In contrast, data from low- and middle-income countries remain more limited, despite the potentially higher burden of pediatric burns in these settings due to environmental risk factors, reduced supervision, and limited access to prevention programs and acute care. This represents a critical gap in the global understanding of pediatric burn epidemiology.

Furthermore, the retrospective design of many studies limits the accuracy and completeness of the data, particularly in relation to the circumstances surrounding injuries, such as supervision levels, environmental context, and socioeconomic status. Low- and middle-income countries remain notably underrepresented in pediatric burn research, despite often experiencing the highest prevalence of such injuries, accompanied by considerable variability in contributing contextual factors.

Finally, there is a lack of standardized protocols for assessing and managing burns in this specific age group, which complicates comparisons across studies and limits the generalizability of the findings. Above all, standardized rehabilitation protocols in the pediatric population are poorly described, further hindering the development of evidence-based guidelines for this vulnerable group. Furthermore, research on the long-term psychological, developmental, and functional effects of burns in early childhood is limited, hindering our understanding of the full impact of these injuries and the effectiveness of interventions over time. Addressing these gaps is crucial for developing more effective prevention strategies and age-appropriate care models.

## 10. Conclusions

Burns in children under 6 years of age represent a significant clinical and preventive challenge, with physical, psychological, and social implications. Preventive interventions at the household and community levels have proven effective in reducing burn incidence. However, significant limitations remain, as most studies lack age-specific analyses, rely on retrospective data, underrepresent low-resource settings, and use heterogeneous or non-standardized protocols. Establishing and strengthening multicenter and multinational burn registries, together with fostering collaborative research networks, would enable the collection of larger and more representative datasets, support the identification of context-specific risk factors, and drive the development of standardized prevention and treatment protocols adaptable to diverse healthcare environments.

## Figures and Tables

**Table 1:** Children and adults burn management: key physiological differences.

Physiological Differences	Pediatric Burns	Adult Burns	Practical/Clinical Impact
Body-surface-area (BSA) to mass ratio and fluid needs	• Much higher BSA-to-weight ratio (e.g., newborn head ≈ 18% TBSA). • Resuscitation threshold ≥ 10% TBSA. • Parkland/other formulas must be modified and maintenance fluids added. • Greater evaporative heat and fluid losses.	• Lower BSA-to-weight ratio (head ≈ 9%). • Resuscitation threshold ≥ 15% TBSA. • Standard adult Parkland formula (no routine maintenance fluids).	• Calculate TBSA with Lund–Browder chart in children. • Avoid over- or under-resuscitation and monitor urine output (≥1 mL kg^−1^ h^−1^ in children; ≥0.5 mL kg^−1^ h^−1^ in adults).
Skin thickness and burn depth	• Thinner epidermis/dermis → deeper injuries from shorter exposure. • Fewer residual keratinocytes → slower re-epithelialization for same contact.	• Thicker skin → greater initial protection. • Identical thermal exposure in adults often produces more superficial burns.	• Higher need for early excision and grafting in pediatric full-thickness burns. • Aggressive scar-modulation (pressure, silicone, steroids, etc.) → risk of hypertrophic scars/contractures even after superficial burns.
Circulatory reserve and blood volume	• Smaller absolute circulating volume → shock develops faster.	• Larger reserve → tolerated volume loss is proportionally less critical.	• Vigilant hemodynamic monitoring → small absolute fluid errors have bigger consequences in children.
Immune maturity and infection risk	• Immature innate and adaptive immunity. • Infection and sepsis more common (≈47% of pediatric burn deaths).	• Fully developed immune responses. • Infection still major risk, but relatively lower incidence.	• Early wound closure, strict asepsis, tetanus status review. • Broad-spectrum antibiotics for suspected sepsis. • Especially in children consider immunonutrition (zinc, selenium, and glutamine).
Thermoregulation and metabolic response	• High surface-area-to-volume, little subcutaneous fat, immature shiver/sweat. • Prone to hypothermia during resuscitation and procedures. • Hypermetabolic surge often exaggerated. • Increased caloric/protein demand.	• More efficient temperature control → hypothermia still possible but less rapid.	• Warm environment and fluids, thermal blankets. • Monitor core temperature closely. • Pharmacologic modulation (e.g., propranolol and oxandrolone) may blunt pediatric hypermetabolism in large burns.
Airway and anatomical considerations	• Smaller airways, larger tongue, proportionally bigger head.	• Larger absolute airway diameter, predictable anatomy.	• In children lower threshold to intubate if inhalation injury is suspected. • Positioning challenges during procedures in pediatric patients.
Psychosocial aspects and rehabilitation	• Developmental stage affects coping → visible scars impact self-image during growth.	• Adult coping mechanisms more mature, but body image concerns are still important.	• Multidisciplinary care with play therapy, long-term scar management, family support; anticipate growth-related contractures needing revision.

Arrow (→) is used to show a cause-and-effect relationship or logical consequence.

## Data Availability

No new data were generated or analyzed in this study.
